# Frequent Alzheimer’s disease neuropathological change in patients with glioblastoma

**DOI:** 10.1093/noajnl/vdae118

**Published:** 2024-07-09

**Authors:** Lisa Greutter, Yelyzaveta Miller-Michlits, Sigrid Klotz, Regina Reimann, Karl-Heinz Nenning, Stephan Platzek, Elena Krause, Barbara Kiesel, Georg Widhalm, Georg Langs, Bernhard Baumann, Adelheid Woehrer

**Affiliations:** Center for Medical Physics and Biomedical Engineering, Medical University of Vienna, Vienna, Austria; Department of Neurology, Division of Neuropathology and Neurochemistry, Medical University of Vienna, Vienna, Austria; Comprehensive Center for Clinical Neurosciences and Mental Health – C^³^NMH, Medical University of Vienna, Vienna, Austria; Department of Neurology, Division of Neuropathology and Neurochemistry, Medical University of Vienna, Vienna, Austria; Comprehensive Center for Clinical Neurosciences and Mental Health – C^³^NMH, Medical University of Vienna, Vienna, Austria; Department of Neurology, Division of Neuropathology and Neurochemistry, Medical University of Vienna, Vienna, Austria; Comprehensive Center for Clinical Neurosciences and Mental Health – C^³^NMH, Medical University of Vienna, Vienna, Austria; Institute of Neuropathology, University Hospital Zurich, Zurich, Switzerland; Center for Biomedical Imaging & Neuromodulation, The Nathan S. Kline Institute for Psychiatric Research, New York City, New York, USA; Department of Neurosurgery, Medical University of Vienna, Vienna, Austria; Comprehensive Center for Clinical Neurosciences and Mental Health – C^³^NMH, Medical University of Vienna, Vienna, Austria; Department of Neurology, Division of Neuropathology and Neurochemistry, Medical University of Vienna, Vienna, Austria; Comprehensive Center for Clinical Neurosciences and Mental Health – C^³^NMH, Medical University of Vienna, Vienna, Austria; Department of Neurosurgery, Medical University of Vienna, Vienna, Austria; Comprehensive Center for Clinical Neurosciences and Mental Health – C^³^NMH, Medical University of Vienna, Vienna, Austria; Department of Neurosurgery, Medical University of Vienna, Vienna, Austria; Comprehensive Center for Clinical Neurosciences and Mental Health – C^³^NMH, Medical University of Vienna, Vienna, Austria; Department for Biomedical Imaging and Image-guided Therapy, Medical University of Vienna, Vienna, Austria; Center for Medical Physics and Biomedical Engineering, Medical University of Vienna, Vienna, Austria; Department of Neurology, Division of Neuropathology and Neurochemistry, Medical University of Vienna, Vienna, Austria; Comprehensive Center for Clinical Neurosciences and Mental Health – C^³^NMH, Medical University of Vienna, Vienna, Austria

**Keywords:** Alzheimer’s disease, amyloid beta, brain aging, glioblastoma, hyperphosphorylated tau

## Abstract

**Background:**

The incidence of brain cancer and neurodegenerative diseases is increasing with a demographic shift towards aging populations. Biological parallels have been observed between glioblastoma and Alzheimer’s disease (AD), which converge on accelerated brain aging. Here, we aimed to map the cooccurrence of AD neuropathological change (ADNC) in the tumor-adjacent cortex of patients with glioblastoma.

**Methods:**

Immunohistochemical screening of AD markers amyloid beta (Abeta), amyloid precursor protein (APP), and hyperphosphorylated tau (pTau) was conducted in 420 tumor samples of 205 patients. For each cortex area, we quantified ADNC, neurons, tumor cells, and microglia.

**Results:**

Fifty-two percent of patients (*N* = 106/205) showed ADNC (Abeta and pTau, Abeta or pTau) in the tumor-adjacent cortex, with histological patterns widely consistent with AD. ADNC was positively correlated with patient age and varied spatially according to Thal phases and Braak stages. It decreased with increasing tumor cell infiltration (*P* < .0001) and was independent of frequent expression of APP in neuronal cell bodies (*N* = 182/205) and in tumor necrosis-related axonal spheroids (*N* = 195/205; *P* = .46). Microglia response was most present in tumor cell infiltration plus ADNC, being further modulated by patient age and sex. ADNC did not impact patient survival in the present cohort.

**Conclusions:**

Our findings highlight the frequent presence of ADNC in the glioblastoma vicinity, which was linked to patient age and tumor location. The cooccurrence of AD and glioblastoma seemed stochastic without clear spatial relation. ADNC did not impact patient survival in our cohort.

## Lay Summary

Brain cancer and Alzheimer’s disease are becoming more common. There are some similarities between glioblastoma, an aggressive brain cancer, and Alzheimer’s disease. The authors of this study wanted to test whether there is any connection between Alzheimer’s disease and glioblastoma. To do this, they examined the brain tissue of 205 patients with glioblastoma to see if there were signs of Alzheimer’s disease, (beta-amyloid, amyloid precursor protein, and tau). Their results showed that 52% of patients with glioblastoma had signs of Alzheimer’s disease in the brain tissue near their tumors. The signs of Alzheimer’s were more common in older patients, and less common in areas of the brain where there was more tumor.

**Key Points**

- ADNC is present in glioblastoma-adjacent cortex of 52% of patients.
- ADNC increases with patient age and varies according to affected brain region.
- Microglia response is highest in the presence of tumor cell infiltration plus ADNC further modulated by patient age and female sex.

**Importance of the Study**

We performed a comprehensive screening of Alzheimer’s disease neuropathological change (ADNC) in the tumor-adjacent cortex of a representative cohort of patients with glioblastoma. ADNC was present in approximately half of the patients, modulated by age and affected brain region. Our findings support a common cooccurrence of glioblastoma and AD in the brains of elderly people, which has potential implications (1) for research into synergistic effects between tumor biology and neurodegeneration and (2) for patient management with more detailed cognitive assessments in a sense that a subset may benefit from the future addition of AD-directed therapy to defer cognitive decline.

Aging is a hitherto inevitable biological process that leads to the deterioration of human tissues including the brain, rendering it susceptible to disorders like brain cancer and neurodegeneration.

Glioblastoma, IDH-wild type, CNS WHO grade 4 is the most common and fatal primary brain tumor, whose incidence peaks in the elderly.^1,2^ Age is also the strongest determinant of outcome with elderly patients having the worst median survival of about 8 months.^3,4^ Despite the high clinical relevance of age, the contributions of an aged brain environment to the disease progression and treatment response remain poorly understood.

Alzheimer’s disease (AD) is the most common neurodegenerative disease, which sporadically affects people above age 65.^5,6^ Pathophysiologically, an altered proteostasis with the accumulation of misfolded amyloid beta (Abeta) and phosphorylated tau (pTau) proteins mediate neuronal loss and cognitive decline.^7,8^ Neuropathologically, AD is characterized by the combination of pTau-positive neurofibrillary tangles (NFTs) and Abeta-positive plaques and occasional vascular deposits. Of note, isolated pTau deposits are not limited to AD but are seen in other tauopathies eg, primary age-related tauopathy.^9^

Both disorders are associated with the activation of microglial cells, which were postulated to contribute to disease progression and potentially also initiation.^10–12^ Beyond microglia, preclinical studies suggested direct biologic links, which involve the activation of the WNT pathway, and the secretion of factors by tumor cells such as impL2 or CD44 that impair mitochondrial and synaptic function and induce pTau in neighboring neurons.^13–18^ Only recently, non-tumor-affected brain tissues of patients with glioblastoma were found to display transcriptomic similarities to AD brains converging on accelerated brain aging.^19^

While the cooccurrence of glioblastoma and AD has been histologically documented in small, preliminary series,^20,21^ the exact prevalence of AD neuropathological change (ADNC) in the glioblastoma vicinity remains unknown. Here, we address this question by leveraging a representative in vivo patient cohort for the systematic screening of tumor-adjacent cortex, providing evidence for frequent comorbidity of both disorders most pronounced but not limited to the oldest patient population.

## Material and Methods

### Tissues and Patients

**Figure 1.**
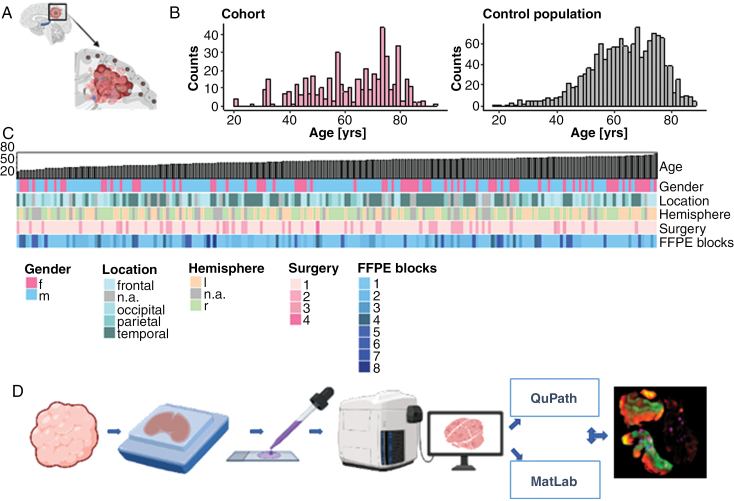
Cohort demographics and workflow. (A) Schematic illustration of glioblastoma-adjacent cortex displaying ADNC. (B) Age distribution of study cohort as compared to the nationwide patient population (*N*_study-cohort_ = 205, *N*_nation-wide_ = 1420). (C) Heatmap detailing clinical factors sorted by ascending patient age. (D) Workflow of tissue embedding, histological processing, image digitization, segmentation, automated quantification, statistical analysis, and data visualization. Created with BioRender.com.

A total of 420 formalin-fixed and paraffin-embedded tissue blocks of 205 patients with glioblastoma, IDH-wild type, CNS WHO grade 4 were included. Additionally, 8 whole brain autopsies from glioblastoma patients were examined. The patients were diagnosed between 2002 and 2022. All tissues were retrieved from the neurobiobank of the Medical University of Vienna with the main inclusion criterion being the presence of a tumor-adjacent cortex in the surgical specimen (Figure 1A). This study was conducted in accordance with the ethical standards outlined in the Declaration of Helsinki. Ethical approval was obtained from the Institutional Review Board at Medical University of Vienna (EK 1429/2022, EK 1375/2018), and written informed consent was obtained from all participants. Patient age ranged from 27 to 92 years with a median age of 67 years, which was representative of the underlying Austrian patient population (median age 64.4 years, based on 1421 patients diagnosed from 2013 to 2018, Figure 1B). The female-to-male ratio was 0.58. The most common tumor location was temporal (*N* = 79/205, 39%), followed by frontal (*N* = 50/205, 24%), parietal (*N* = 26/205, 13%), and occipital (*N* = 24/205, 12%) with a preference for the right hemisphere (ratio 1.28). The lobar location was not available for 26 patients, and information on the hemisphere was lacking in 41 patients. A total of 16 patients had longitudinal samples across 2 to 4 time points. The cohort demographics are summarized in Figure 1C.

### Tissue Processing

An overview of the workflow is presented in Figure 1D. In brief, formalin-fixed and paraffin-embedded blocks were cut at a thickness of 2 µm followed by immunohistochemistry using the following antibodies: Anti-NeuN (EMD Millipore, USA, clone MAB377, 1:2000, mouse, heat-induced epitope retrieval [HIER] pH6), anti-b-A4 (DAKO, Denmark, clone 6F/3D, 1:100, mouse, 80% formic acid [FA] 1 hour), anti-t-AT8 (Invitrogen, Belgium, 1: 200, clone AT8 pS202/pT205, mouse, no pretreatment), anti-APP (EMD Millipore, USA, clone 22C11, 1:8000, mouse, HIER pH6), anti-Iba1 (Wako, Japan, 1:1000, rabbit, HIER pH6), anti-CD68 (DAKO, Denmark, clone KP1, 1:5000, mouse, HIER pH6), anti-HLA-DR (DAKO, Denmark, clone CR3/43, 1:400, mouse, HIER pH6), anti-tau-RD3 (EMD Millipore, USA, clone 8E6/C11, 1:16000, mouse, HIER pH6 plus FA 100% 1 minute), and anti-tau-RD4 (EMD Millipore, USA, clone 1E1/A6, 1:800, mouse, HIER pH6 plus FA 100% 1 minute). Immunostaining was performed on the Leica DAKO Autostainer Link 48 with the DAKO Envision System Kit (DAKO, Glostrup, Denmark) for mouse- and rabbit-anti-human primary antibodies using diaminobenzidine as chromogen. The incubation time of the primary antibodies was 30 minutes for anti-t-AT8, anti-tau-RD3, anti-tau-RD4, anti-APP, anti-CD68, and anti-HLA-DR, 60 minutes for anti-b-A4, and 2 hours for anti-Iba1 at room temperature. Slides were mounted and digitized using a Hamamatsu NanoZoomer 2.0 HT slide scanner at a magnification of 40X. Double immune fluorescence stainings with APP and Nestin as well as APP and NeuN were performed to validate the APP expression in neurons and tumor cells. OPAL 690 and OPAL 620 were used as fluorescence markers, APP and NeuN were used as described above. Nestin (Millipore, USA, clone 10C2, 1:250, mouse, HIER pH6, incubation time 60 minutes) and NeuN were labeled with OPAL 690, APP with OPAL 620. The slides were mounted and digitized using Akoya Vectra Polaris.

Eight postmortem brains of individuals with glioblastoma were analyzed (median age 65.5 years). In each case, ground truth AD stages were assessed based on the following regions: hippocampus, middle and superior temporal gyrus, middle frontal gyrus, parietal med/inf gyrus, occipito-medial cortex, basal ganglia, thalamus, pons, medulla oblongata, cerebellum. In addition, tumor-adjacent cortex was randomly sampled and included the following regions: Frontal cortex (*N* = 4), parietal cortex (*N* = 2), and insular cortex (*N* = 2). All blocks were cut and stained for anti-b-A4 and anti-t-AT8. The median Braak & Braak stage was II (range 0–V), the median Thal phase was 2 (range 0–5), and the median CERAD score was A (range 0–C). Notably, one patient exhibited no Alzheimer’s disease-related changes.

### Semiquantitative Scoring

In whole slide scans, cortex and tumor tissues were manually segmented using the segmentation functionality of QuPath.^22^ Using NeuN stainings, cortical tumor cell infiltration was graded as low, medium, and high with medium showing an approximately equal ratio of neurons to nonneuronal cells (1:1), and high showing more than 70% nonneuronal cells. Abeta plaque density was scored based on CERAD scores (0 = diffuse plaques only; A = 1–3 neuritic plaques per high-power field); (B = 5–10 neuritic plaques; C = frequent [> 20] neuritic plaques). Cerebral amyloid angiopathy (CAA) was recorded as present or absent in blood vessels and in capillaries. pTau accumulation in neurons was scored according to Braak & Braak stages into 4 categories (0 = negative; I to II = NFTs in hippocampal regions; III to IV = NFTs, threads, and dots in temporal neocortex; and VI to V = NFTs, threads and dots in frontal to occipital neocortical regions). To quantify the severity of ADNC, the scores from both staging systems were combined. All ADNC-related parameters were independently scored by 2 neuropathologists (SK, RR). Cohen’s Kappa was calculated to assess inter-rater agreement. APP was semi-quantitatively assessed for its expression by tumor cells, neurons, and within axonal spheroids highlighting diffuse axonal injury (DAI). Cellular expression was scored as sparse (1% to 5% of cells), moderate (10% to 30%), and abundant (more than 50%). The presence of axonal spheroids was classified as absent, sparse (1–10 per high-power field in hot spots), moderate (10–30), and frequent (>30). Representative histological images of semiquantitative scores are provided in Supplementary Figure S1.

### Automated Quantification

In the first step, NDP.view2 software (Hamamatsu) was used to annotate cortical regions as well as to define the outline of whole slide scans. In QuPath, the densities of NeuN, Iba1, CD68, and HLA-DR-positive cells were quantified using the “positive cell count” function for NeuN and the “pixel classification” for CD68, HLA-DR, and Iba1 across the entire whole slide scans. Values were then exported to R for further statistical analysis.^23^ In a second step, the neuronal, nonneuronal, and microglial cell densities, Abeta density, pTau density were analyzed in a pixel-wise, coregistered way using custom code in MATLAB (R2018b, The MathWorks). Therefore, whole slide scans were exported as JPG files using the 10X compression setting. To account for the variability of staining intensity between different slides, a graphical user interface was implemented. For each image, 10 randomly selected pixels displaying immunostained structures as well as 10 pixels with hematoxylin-stained nuclei were manually marked and the average RGB color vectors of immunostain and hematoxylin were stored for further segmentation tasks. Slide scan images were downsampled by 0.5 × 0.5 to speed up computational tasks while maintaining sufficient resolution for segmentation. Binary tissue on background masks was generated by thresholding each slide scan image at the mean level of its blue color channel and using the “imerode” and “imdilate” functions of MATLAB. Next, the angle between the RGB color vector of each pixel of the slide scan and the 2 respective RGB color vectors (eg, hematoxylin and NeuN immunostain) were computed from their dot product to estimate the alignment of a pixel’s color with the average color vectors of the 2 stains. Based on these angles and 2 empirically found thresholds, each pixel of the slide scan was classified as hematoxylin stained, immunostained, or unstained. Hematoxylin-stained cell nuclei were segmented by feeding the classification result into an additional thresholding step and morphological operations. Specifically, the vector products of a slide scan image and the respective average RGB vector of hematoxylin-stained pixels were normalized and thresholded at 30%, followed by image erosion (“imerode” using a disk with a radius of 1 pixel). Next, the centroids of the segmented cells were localized and cell density maps were computed by convolving the centroid maps with a circular kernel (structured element “disk” in MATLAB) with a radius of 200 pixels, corresponding to an area of 0.42 mm². A similar process was used to segment the immunostained pixels using a threshold of 40%. Again, maps displaying the centroids of segmented particles were convolved with a similarly sized “disk” to compute heatmaps of immunostained particles such as NFTs or plaques. The NDPA files containing the annotation polygons from NDP.view2 were renamed to XML format and the slide scan scaling was inferred from the manually drawn bounding boxes/slide outlines in NDP.view2. The annotation polygons were used to generate images containing binary cortex and tumor masks. For each sample, the heat maps displaying the density of immunostained particles, the corresponding cell density maps from the hematoxylin channel, and the respective binary masks (tissue on background, cortex) were loaded and downsampled by another 0.0125 × 0.0125. Each pixel in these downsampled maps corresponds to an area of 73 × 73 µm = 5329 µm². The heat maps and binary masks were aligned with respect to the NeuN slide by rigid image registration (MATLAB function “imregister”) using the background maps as a backbone. Finally, for all pixels representing cortical tissue (ie, tissue-on-background map = 1 and cortex = 1), the density data of the aligned heat maps was stored as row vectors representing tumor cell density (per mm²) from the hematoxylin channel of the NeuN slide, cell density (per mm²) of the NeuN channel of the NeuN slide, Abeta density (particles per mm² where one particle roughly corresponded to one plaque or vessel), pTau density (particles per mm² where one particle corresponded to one NFT, while grains and threads were not counted), Iba1 density (particles per mm² where one particle corresponded to one microglial cell). For samples without Abeta/pTau deposits, Abeta and pTau heatmaps were not computed. Representative examples of the segmented cells and structures are shown in Supplementary Figure S2.

### Group-Level Statistical Analysis and Data Visualization

Statistical and graphical analysis was performed in R-Studio (version 1.4.1106). A Shapiro–Wilk test was employed to assess the presence of a normal distribution (P-value > .05). ANOVA followed by pairwise t-tests with Bonferroni correction were employed to compare the means among groups. Data were visualized using violin plots. To predict the P-values for correlations involving normally distributed variables, Pearson’s correlation was employed, while Kendall’s tau was utilized for variables that were not normally distributed. The linear models were performed using Pearson’s correlation. For the statistical analysis of cross tables, Fisher’s exact test was implemented. Cox regression analysis with Kaplan–Meier plot visualization was used to explore the impact on survival. To ensure robustness, the alpha value was set at a threshold of 0.05. Heat maps were generated with MATLAB and ImageJ/Fiji,^24^ histological pictures were exported using Halo software v3.6.4134.166 (Indica Labs, Albuquerque NM) and NDPI-Viewer.

## Results

### Cortical Tumor Cell Infiltration is Associated With Loss of Neurons

**Figure 2.**
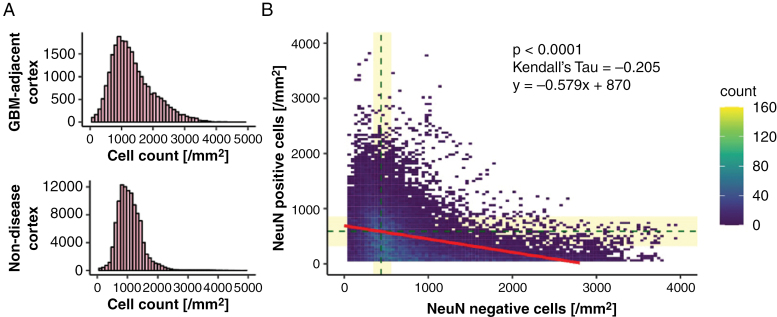
Cortical tumor cell infiltration is associated with neuronal loss. (A) Cell counts per mm^2^ in glioblastoma-adjacent cortex (upper panel, median = 1221 cells/mm²; *N* = 205 individuals) and in non-diseased cortex of age-matched postmortem brains (lower panel, median = 1030 cells/mm², *N* = 5 control brains, *P*-value < .0001). (B) Scatter plot illustrating the distribution of NeuN-positive neurons (on the *y*-axis) and NeuN-negative nonneuronal cells in the cortex. Median values obtained from 3 postmortem controls are indicated by horizontal and vertical dashed lines, while the bars indicate the confidence intervals (Kendall’s tau = −0.205, *P*-value < .0001, *N* = 205).

A median of 35.8 mm² (range 1 to 1150 mm²) of cortex was present per specimen with regions of low, medium, and high tumor infiltration often present in the same slide (Supplementary Figure S3A). The age of the patient did not impact the extent of the cortical area (Supplementary Figure S3B). Overall, the median cell density in tumor-adjacent cortex was 1221 cells/mm², which was significantly higher as compared with control brains (median 1030 cells/mm², *P*-value < .0001, Figure 2A). Total cell counts were similar across cerebral lobes and hemispheres, among females and males, and across age groups (Supplementary Figure S3). Overall, the number of neurons decreased with increasing counts of nonneuronal cells (Kendall’s tau = −0.205, *P*-value < .0001, Figure 2B).

### Frequent ADNC in Glioblastoma-Adjacent Cortex

**Figure 3.**
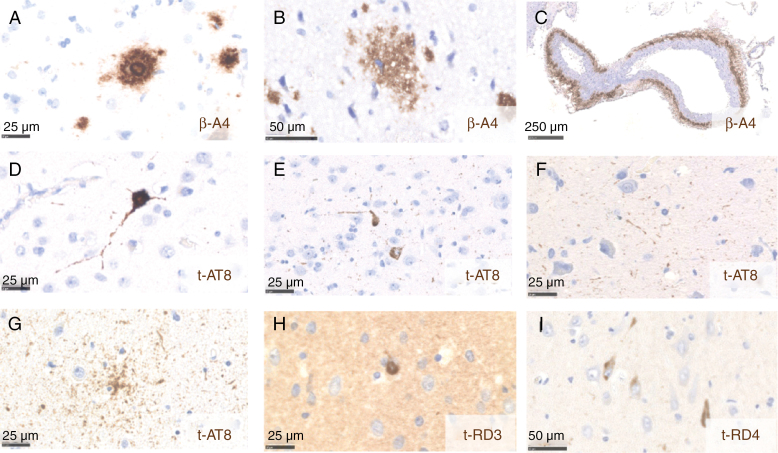
Representative phenotypes of ADNC in tumor-adjacent cortex. (A) Neuritic plaque (scale bar = 25 µm). (B) Diffuse plaque (scale bar = 50 µm). (C) CAA (scale bar = 250 µm). (D) Neurofibrillary tangle (scale bar = 25 µm). (E) Pretangle (scale bar = 25 µm). (F) Neuropil threads (scale bar = 25 µm). (G) Granular fuzzy astrocyte (scale bar = 25 µm). (H) 3R immunoreactive neuron (scale bar = 25 µm). (I) 4R immunoreactive neuronal pathology (scale bar = 50 µm).

**Figure 4.**
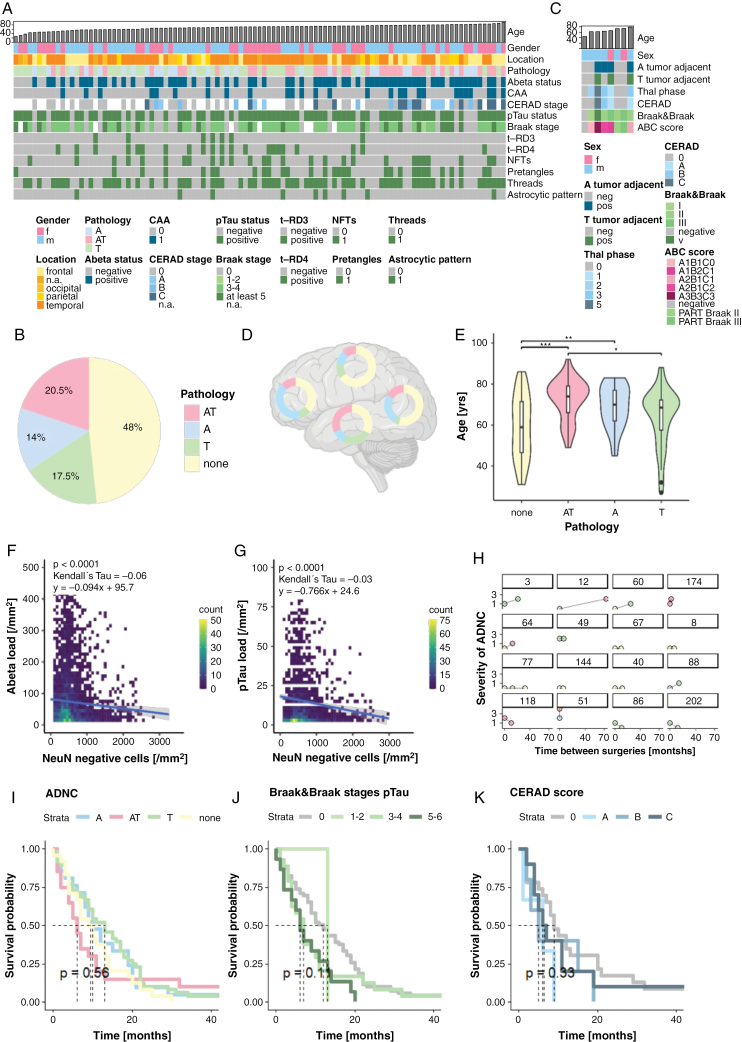
Neuropathological mapping of ADNC. (A) Heatmap of Abeta (A) and pTau (T) deposits per patient sorted by age (*N* = 106). (B) Distribution of ADNC (*N* = 205). (C) Heatmap of a postmortem glioblastoma cohort (*N* = 8) with ADNC ratings for random tumor-adjacent cortex in comparison to ground truth Braak & Braak stages, Thal phases, and CERAD scores. (D) Topographic differences among ADNC patterns (*N* = 179). (E) Boxplot of the age range per protein deposits (*N* = 205). (F) 2D-histogram of Abeta load against tumor cell density in the cortex (*N* = 66, *P*-value < .0001, Kendall’s tau = −0.06). (G) 2D-histogram of pTau load against tumor cell density in the cortex (*N* = 77, *P*-value < .0001, Kendall’s tau = −0.03). (H) Distribution of pathology type (refer to color-scheme of B, C, and D) and load among primary and recurrent glioblastoma (*N* = 16). To assess the concomitant ADNC load, Braak & Braak stages and CERAD scores were combined. (I) Kaplan–Meier analysis stratified according to ADNC status (*N* = 133, *P*-value = .56). (J) Kaplan–Meier analysis stratified into Braak & Braak stages (*N* = 133, *P*-value = .11). (K) Kaplan–Meier analysis stratified according to CERAD score (N = 133, *P*-value = .33).

Neuropathologically, Abeta deposits were noted as neuritic or diffuse plaques, and in vessel walls, while pTau deposits were found as NFTs, pretangles, threads, and glial granular fuzzy astrocytes. Representative phenotypes are provided in Figure 3. Overall, 52% of the patients (N = 106/205) showed ADNC in tumor-infiltrated cortex with substantial between- and within-subject variability (Figure 4A). Twenty percent of patients (*N* = 41/205) had combined Abeta and pTau (AT) deposits, while 18% (*N* = 37/205) had pTau (T) deposits only, 11% (*N* = 22/205) Abeta plaques (A) only, and 3% (*N* = 7/205) sole vascular Abeta (Figure 4B). Among the latter, 17 cases displayed capillary CAA (Allen type 3, Thal type 1). Among samples with isolated pTau deposits (*N* = 41), 24 samples were screened for 3 and 4 repeat tau isoforms with 8 having both isoforms, consistent with ADNC, and 16 cases displaying 4 repeat tau only (Supplementary Figure S4A).^25^ Among the 16 cases, 9 showed pTau deposits in glial cells (*N* = 8) and grains (*N* = 1), indicative of another 4R tauopathy and were removed for further ADNC-related analyses. pTau and Abeta were independently assessed and scored by 2 observers (SK and RR) with high to excellent inter-rater agreement (Cohen’s Kappa scores from 0.673 to 1, Supplementary Figure 4 and Supplementary Table 1).

Overall, the spatial distribution followed endogenous patterns of AD progression according to Thal phases and Braak stages (scheme provided in Supplementary Figure S4B, C)^26–28^ with the highest density of NFT and neuropil threads in the early affected temporal cortex, and the highest Abeta density in the occipital cortex (Figure 4C). ADNC varied by patient age with Abeta and combined deposits being significantly more prevalent in elderly patients (Figure 4D). The prevalence of ADNC did not differ between females and males (Supplementary Figure S4D, *P*-value = .39) and according to MGMT gene promoter methylation status (Supplementary Figure S4E, *P*-value = .8).

When taking putative Braak & Braak stages into account, the majority of individuals fell into medium or high stages (stages III-IV *N* = 50; stages V-VI N = 19, Figure 4A). In contrast, many patients had early CERAD plaque scores (score 0: *N* = 38; score A: *N* = 6) with fewer displaying scores B (*N* = 8) and C (*N* = 13). Logistic regression revealed that CERAD scores were significantly associated with age and Braak & Braak stages (*P*-value_age_ < .0001, *P*-value_Braak & Braak_ < .0002), while Braak & Braak stages were associated with temporal and parietal location, and CERAD score (*P*-value_temporal_ < .0001; *P*-value_parietal_ = .0346; *P*-value_CERAD_ = .0002, Supplementary Figure 4G, H). The association between Braak & Braak stages and brain location was confirmed in the subgroup of individuals <60 years (*P*-value_temporal_ < .0001; Supplementary Figure 4I) but no longer present in the few patients aged <40 years. In an attempt to better characterize the sampling error due to random cortical tumor locations, we leveraged a postmortem cohort of patients with glioblastoma with ground-truth ADNC. Random tumor-adjacent cortical samples demonstrated concordant Abeta results in 7 of the 8 patients, while pTau was less consistently sampled (detected in 3 of the 7 patients) with early Braak stages I-II being largely undetected (0 of the 4 cases, Figure 4C). Despite this caveat, direct comparison with 12 major population-based studies (PMIDs included in Supplementary Figure 4J) suggested the observed Abeta frequencies to be within normal age ranges, while pTau exceeded age-matched controls in younger individuals only (CAVE only 1 population-scale study in younger individuals, which used silver stainings to differentiate pretangles from NFT, Supplementary Figure 4J&K).

Higher Abeta and pTau load was observed in the absence or during low-level cortical tumor cell infiltration, while more extensive infiltration was associated with decreased Abeta and pTau deposits (Figure 4, F and G; A: *P*-value < .0001, Kendall’s tau = −0.06; T: *P*-value < .0001, Kendall’s tau = −0.03). For 16 individuals (median age 67 years) matched recurrent tumors plus adjacent cortex were available with a median time between first and second surgery of 10.2 months. Five cases demonstrated an increase of ADNC, while the remainder had stable pathology (*N* = 7), fewer deposits (*N* = 5), or altered protein patterns, eg, transitioning from Abeta and pTau to only pTau (*N* = 6, Figure 4H). The median time to second surgery was highest in those patients, who showed an increase in ADNC (25.8 vs. 10.4 months). For 8 patients with available treatment information, ADNC did not correlate with type of treatment (χ²_CT_ = 1.6, *P*-value_CT_ = .45; χ²_RT_ = 1.1, *P*-value_RT_ = .56; χ²_combTX_ = 1.6, *P*-value_combTX_ = .44, Supplementary Table S2).

In the entire cohort, the presence of ADNC was not associated with patient survival (log rank test *P*-value = .58). A nonsignificant trend towards worse outcomes for cases with combined pathology (*N* = 133, *P*-value_ADNC_ = .56), high CERAD (*P*-value_CERAD_ = .33) score and advanced Braak & Braak stage (*P*-value_Braak & Braak_ = .11, Figure 4I–K) was observed. Stratification into age cohorts revealed ADNC to be associated with worse outcomes in younger patients aged <50 years (Supplementary Figure 5B–D, *P*-value_<50_ = .07, *N* = 26). Likewise, when stratifying according to MGMT promoter methylation status, ADNC and Braak & Braak stages were associated with worse survival in both subgroups (Supplementary Figure 5J–M; *P*-values from .017 to .049). The CERAD score was associated with worse outcome only in the subset of unmethylated tumors (Supplementary Figure 5N, O; *P*-value_CERADunmethMGMT_ = .0097).

### Microglia Activation in the Presence of Isolated and Dual Pathology

**Figure 5.**
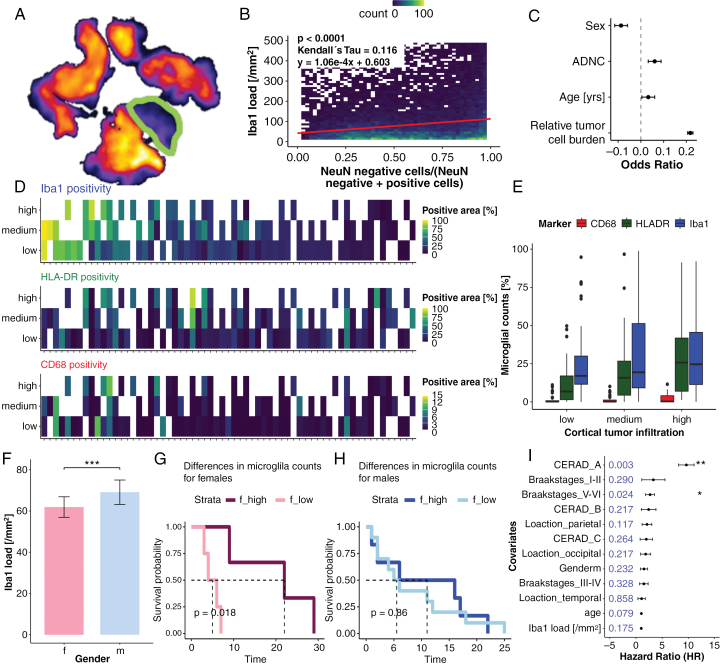
Microglia activation in dual pathology. (A) Heatmap illustrating microglial activation in tumor and cortical tissue (encircled). (B) 2D-histogram for the association between Iba1 and tumor cell burden (*N* = 205, Kendall’s tau = 0.116, *P*-value < .0001). (C) Forrest plot of Pearson correlations for the effect of each variable on Iba1 count per mm². Female sex was associated with significantly higher microglial counts (*N* = 205). (D) Association among CD68, HLA-DR, and Iba1 as assessed in relation to cortical tumor cell infiltration (*N* = 78). The *x*-axis corresponds to individual samples. Colors correspond to cell counts with white boxes indicating a lack of cortex with respective tumor cell infiltration. (E) Boxplots of microglial counts according to cortical tumor cell infiltration (*N* = 78, nonsignificant increases). (F) Differences in microglial activation between males and females are displayed as a box plot (*P*-value < .00001, *N* = 205). (G) Kaplan–Meier plot for differences in overall survival for females between individuals with high and low microglial activation (*P*-value = .018, *N* = 15). (H) Kaplan–Meier plot for males with high and low microglial activation (*P*-value = .86, *N* = 35). (I) Multivariate Cox regression analysis for the impact of different ADNC-related and unrelated parameters on overall survival. *P*-values are highlighted, statistical significance is indicated by * (*N* = 133).

Overall, microglia counts were magnitudes higher in the tumor as compared to tumor-adjacent cortex (Figure 5A). Within the cortex, microglia counts increased with increasing tumor cell infiltration (Kendall’s tau = 0.116, *P*-value < .0001, *N* = 205, Figure 5B), which was the most relevant factor, followed by the presence of ADNC and patient age. Conversely, male sex was negatively associated with microglia counts (Figure 5C, *N* = 205, Supplementary Figure S6A, B). Microglia counts differed significantly between males and females (*P*-value < .00001), and stratification at the sex-specific median resulted in significant survival differences only in females (worse outcome in presence of high microglia counts, univariate *P*-value = .018, Figure 5G). The independent effect of microglia counts on survival was not confirmed upon multivariate survival analysis (Figure 5I).

To capture different levels of microglial activation (ie, Iba1 and CD68 for increased phagocytic activity and cell mobility, HLA-DR for secretion of inflammatory cytokines),^29,30^ matched Iba1, HLA-DR, and CD68 stainings were analyzed for a subset of 78 randomly selected cases. Since tumor cell infiltration was the strongest predictor of microglial counts, cortical areas were classified as low, medium, and high tumor cell infiltration. The highest microglia counts were almost exclusively observed in regions with high tumor cell infiltration (*P*-value _low vs high_ < .01, *P*-value _low vs medium_ <.05, *P*-value _medium vs high_ = 1, *N* = 78). Among the different markers, Iba1 was most common, followed by HLA-DR, while CD68 was expressed by fewer cells (all group-wise comparisons *P*-value < .001, Figure 5D, E, *N* = 78).

### Frequent Expression of APP and DAI

**Figure 6.**
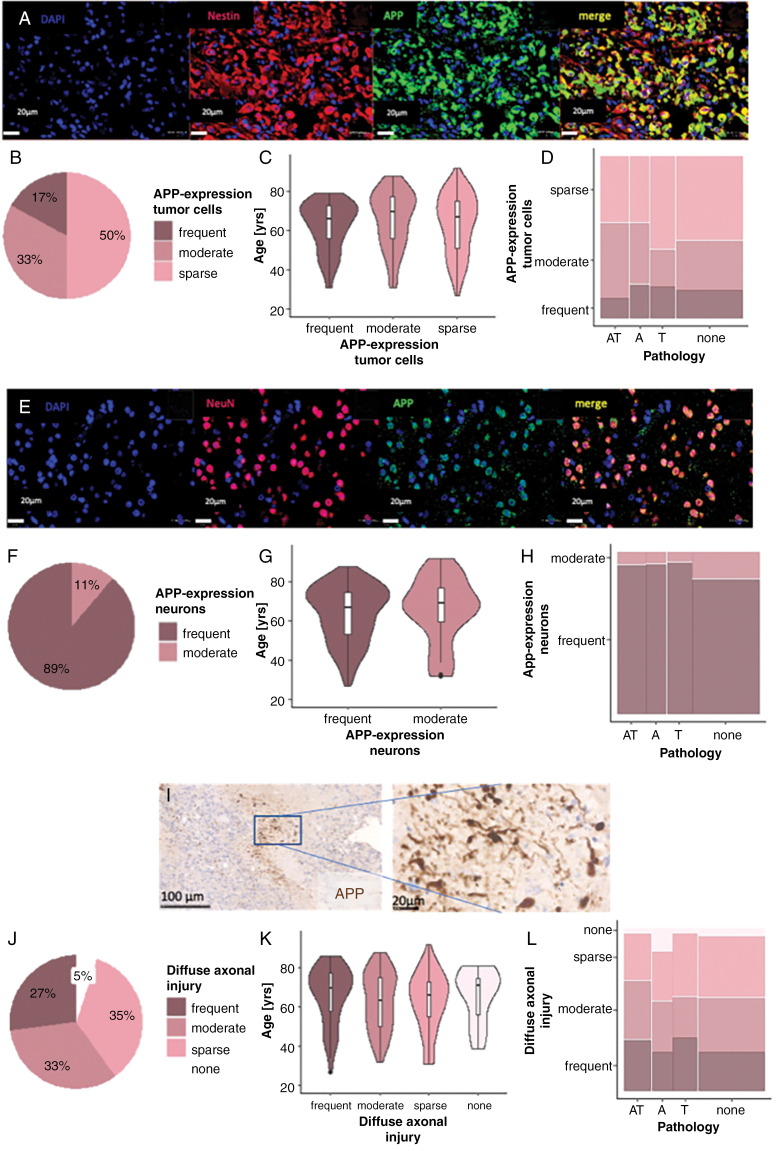
Amyloid precursor protein (APP) expression and diffuse axonal injury (DAI). (A) APP expression by tumor cells (scale bar = 25 µm). Double-staining of APP and Nestin, all scale bars = 20 µm. (B) Prevalence of APP expression in tumor cells. (C) Effect of age on APP expression in tumor cells (*N* = 205, *P*-value = .49). (D) Effect of APP expression by tumor cells on ADNC (*N* = 205, *P*-value = .37). (E) APP expression by tumor-adjacent neurons (scale bar = 25 µm). (E) Double labeling of APP with NeuN, scale bars = 20 µm. Region-of-interests are representative of cases with semiquantitative scores of frequent APP expression (50%–100%). (F) Prevalence of APP expression in neurons (*N* = 205). (G) Effect of age on APP expression in neurons (*N* = 205, *P*-value = .56). (H) Effect of APP expression of neurons on ADNC (*N* = 205, *P*-value = .26). (I) Dense DAI surrounding tumor necrosis (scale bar = 100 µm). (J) Prevalence of DAI (*N* = 205). (K) Effect of age on DAI (*N* = 205, *P*-value = .55). (L) Effect of DAI on ADNC (*N* = 205, *P*-value = .46).

Amyloid precursor protein (APP), a precursor molecule, whose cleavage generates Abeta, was expressed in 50% of the tumors with 17% expressing it in >50% of the tumor cells (Figure 6, A and B). APP expression did neither correlate with patient age (Figure 6C, *P*-value = .49), nor the presence and severity of ADNC (Figure 6D, *P*-value = .37). In all cases, APP was also expressed by tumor-adjacent neurons with 89% expressing it in more than 50% (Figure 6, E and F). APP expression in tumor cells and neurons was confirmed upon double labeling of APP with Nestin and APP with NeuN (Figure 6, A and E), and the semiquantitative ratings were confirmed by quantifications of double-labeled cells across 3 regions of interest per case. There was no significant difference in the median age of patients with frequent or moderate neuronal APP (Figure 6G, *P*-value = .56) expression and APP expression did not correlate with ADNC (Figure 6H, *P*-value = .26).

APP accumulation in axons, which is typically detectable from 2 to 3 hours until up to 9 months after axonal damage,^31^ was mostly present in and around tumor necrosis, commonly as a zigzag pattern consistent with an ischemic/hypoxic genesis (Figure 6I and Supplementary Figure S7). DAI was frequent in 27% (*N* = 55/205) of the cases and absent in 5% (Figure 6J). It was independent of patient age (Figure 6K, *P*-value = .55), and presence and degree of ADNC (Figure 6L, *P*-value = .46). To evaluate the impact of surgery, epilepsy surgery specimens were evaluated for DAI with 3 out of 4 showing no evidence for DAI.

## Discussion

In the present study, we leveraged a large cohort of patients with glioblastoma to systematically screen tumor-adjacent cortex for ADNC. The most noticeable result was the frequent presence of ADNC, ie, neurofibrillary tangles and/or amyloid plaques in roughly half of all patients albeit fewer patients with severe ADNC load (Braak & Braak stages V-VI, CERAD C). This surprisingly high rate of comorbidity extends previous histology-based studies, which highlighted occasional AD protein deposits in the vicinity of brain tumors.^20,21^ Noteworthy, previous studies suggested that the histological patterns vary by tumor type and behavior. For instance, while slowly growing lesions such as cortex-invading meningiomas or gangliogliomas are mostly associated with isolated pTau deposits,^32–35^ hence rather than suggesting a secondary mechanism,^36^ combined Abeta and pTau deposits, morphologically consistent with AD, were mostly limited to individuals of old age.^20,21^ In line with this notion, in a previous analysis of postmortem brains of patients with glioblastoma we did not observe an accumulation of ADNC at the site of the tumor, which prompted us to consider rather a stochastic cooccurrence than tumor-induced mechanism.^20^ This concept is further confirmed in the present setting, where the degree of cortical tumor cell infiltration was not positively correlated with the severity of ADNC. In fact, the negative correlation of ADNC with tumor infiltration was likely due to progressive destruction of cortex with the loss of neurons and ADNC. Taken together, in older individuals with glioblastoma, Abeta and/or pTau deposits varied according to tumor location but did not exceed population-based controls, whereas excess isolated pTau deposits in younger patients might exceed population controls,^26^ potentially suggesting a tumor-induced secondary mechanism.

All surgical specimens of the present series were derived from neocortical or limbic regions, while no basal ganglia or brainstem gray matter was present. Hence, the present Abeta pathology would at least correspond to Thal phases 1 or 2 with no ability to assess more advanced stages. This is important to keep in mind since Thal phases 1 and 2 are both compatible with cognitively asymptomatic individuals. Likewise, pTau aggregates, which correlate more closely with cognition,^37,38^ were mostly detected in temporal and frontal cortices, hence corresponding to Braak stages III to V. Clinically, one would expect mild cognitive impairment with a higher risk of conversion to dementia in stage VI-V patients.^39^ Given the descriptive nature of the present study, the oftentimes limited area of tumor-adjacent cortex, the lack of imaging/liquid biomarkers as well as pre-tumor cognitive performance status our findings need to be cautiously interpreted and no firm conclusions can be drawn on the clinical impact of ADNC on individual patients. Likewise, in the present cohort, we did not observe consistent associations with survival albeit trends towards worse outcomes for the presence and severity of ADNC, as well as in subgroups stratified according to age and MGMT status. The latter is remarkable since MGMT variants were recently identified as a risk factor for AD in a genome-wide association study.^40^

Interestingly, we found distinct patterns of APP accumulation, which is a direct precursor protein to Abeta, and which was present in tumor-adjacent neurons and axonal spheroids accentuated around areas of tumor necrosis. Both histological patterns are established risk factors for the development of chronic traumatic encephalopathy, which is mostly associated with pTau inclusions in neurons, astrocytes, and neurites.^36,41–44^ Axonal spheroids require 2–3 hours to form after the acute disconnection of axons, and since they were exclusively present in tumor but not epilepsy surgical specimens, we consider them to be linked with tumor necrosis rather than surgical dissection. In the present series, neither APP expression nor DAI was associated with the presence and degree of ADNC. Whether this is a true finding or due to the aggressive disease course of elderly patients with glioblastoma, which prevented us from seeing the mid- to long-term sequelae, remains speculative. As a side note, we also confirmed strong and abundant expression of APP by glioblastoma tumor cells, similar to reports in several cancer types including melanoma metastases, where the expression of APP was a prerequisite for successful brain seeding.^42,45^ With an increasing armamentarium of melanoma-directed drugs and increasing patient survival, it will be interesting to study long-term effects on Abeta aggregation and spread in those patients.

Glioblastoma and AD are in part driven by an altered innate immune response prominently involving microglia.^46–49^ In our cohort, the microglial response to glioblastoma cancer cells was magnitudes higher than to ADNC. Not surprisingly though, concomitant ADNC and high patient age were aggravating factors. Notably, we observed higher microglial counts in females, which is in line with previous work.^50,51^ While the precise molecular, hormonal, and environmental reasons for this discrepancy remain obscure, sex-specific differences in microglia morphology, function, and metabolism with diminished phagocytic activity and fewer MHC II expression in female microglia were previously linked with the progression of glioblastoma and AD.^51,52^ Hence, in future studies, it will be important to explore the transcriptomic and functional status of microglia in sex-specific contexts to elucidate their biological role in multimorbid brains.

Ultimately, treatment-induced accelerated brain aging is an increasingly recognized field with broad implications for many patients receiving chemo- and radiotherapy. Given that many patients with glioblastoma receive intense multimodal treatment and suffer from concomitant ADNC at the time of diagnosis, we hypothesized to find a longitudinal increase of AD load. Due to the limited availability of longitudinal tissues in elderly patients with very few elderly patients undergoing second resections (including tumor-adjacent cortex), the present series was not adequately powered to assess changes in ADNC even though it highlighted an increase in patients with prolonged time to tumor recurrence.

Our study has further limitations. First, the location and extent of cortex amenable to screening depended on tumor location and was insufficient for a complete AD staging with CERAD scores and Thal phases likely being more reliably sampled from random cortical regions than pTau pathology. Second, APOE4 genotype and allele frequency, as one important risk factor for AD, was not available. However, capillary CAA frequencies (Allen type 3, Thal type 1) of the present cohort (8 %) were in the range of reported APOE4 frequencies for European populations.^53,54^ Third, our custom digital image analysis pipeline was based on multiple serial sections per tissue block. While great care was taken to properly register heat maps across different slides, distortions may have led to slight misalignments between corresponding regions and therefore blur our correlative analysis. To compensate for strong staining differences between slides and samples and to obtain the best possible segmentation results, we opted to choose a rather labor-intensive approach using manual marking of stained colors. More sophisticated analysis methods, eg, based on deep learning, will be necessary for the evaluation of larger data sets and also to distinguish subtle structures such as individual neuropil threads.

In conclusion, we report frequent concomitant ADNC in patients with glioblastoma, which followed spatial patterns of AD evolution and correlated with age. A lack of positive correlation with the degree of tumor-cell infiltration suggested rather stochastic cooccurrence. The innate immune response was magnitudes higher to the tumor as compared to cortical AD-type pathology and further modulated by patient age.

## Supplementary Material

vdae118_suppl_Supplementary_Data
