# Supplementary material for: Frequent Alzheimer’s disease neuropathological change in patients with glioblastoma
Source: Neurooncol Adv. 2024 Jul 9;6(1):vdae118. doi: 10.1093/noajnl/vdae118 (PMC11362848; doi:10.1093/noajnl/vdae118)
Supplement: vdae118_suppl_Supplementary_Data [file vdae118_suppl_Supplementary_Data.zip › Supplementary Table and Figure Legends_NOA-D-24-00064R1.docx]

### Supplementary Data

***Supplementary Figure S1.*** *Semiquantitative scoring. (A) Neuronal cell scores (left: low tumor infiltration, middle: medium tumor infiltration, right: high tumor infiltration, scale bar = 50µm). (B) Abeta scores (left: CERAD 0, middle-left: CEARD A, middle-right: CERAD B, right: CERAD C, scale bar = 100µm).* *(C) pTau scores (left: Braak & Braak I-II, middle: Braak & Braak III-IV, right: Braak & Braak V-VI, scale bar = 50µm). (D) APP expression in tumor cells (left: sparse, middle: moderate, right: frequent, scale bar = 50µm). (E) APP expression in neurons (left: moderate, middle: frequent, scale bar = 50µm). (F) Diffuse axonal injury (left: sparse, middle: moderate, right: frequent, scale bar = 50µm).*

***Supplementary Figure S2.*** *Cell segmentation and quantification using custom Matlab code. (A) Whole slide scan of a NeuN stained section with cortex segmentation in blue and tumor segmentation in red. (B) Three representative regions highlighting segmentation of individual neurons by red crosses. The scale bar indicates 50µm. (C) Density map of NeuN-positive cells per mm² in whole slide scan. (D) Whole slide scan of NeuN stained-section with tumor annotation in red and adjacent cortex in blue. (E) Segmentation of hematoxylin-stained cells per mm² in the NeuN staining, identified cells are highlighted with red crosses. The scale bar indicates 50 µm. (F) Density map of non-NeuN labeled cells per mm² in whole slide scan. (G) Whole slide scan of Abeta stained-section with tumor annotation in red and adjacent cortex in blue. (H) Single plaques are identified by red crosses in Abeta staining. Note, the segmentation is more error-prone in case of dispersed and ill-defined Abeta deposits (right image). The scale bar indicates 50µm. (I) Density of Abeta plaques per mm² in whole slide scan. (J) Whole slide scan of pTau stained-section with tumor annotation in red and adjacent cortex in blue. (K) Single NFTs are identified by red crosses. (L) Density of NFTs per mm² in whole slide scan. The scale bar indicates 50µm. (M) Whole slide scan of Iba1 stained-section with tumor annotation in red and adjacent cortex in blue. (N) Single microglia are identified by red crosses in the Iba1 staining. The scale bar indicates 50µm. (O) Density of microglia per mm² in whole slide scan.*

***Supplementary Figure S3.*** *Cortical area and cell counts. (A) Histogram of preexisting cortex in the cohort (N=205, median=35.8 mm²). (B) Correlation of cortical area with age (the blue line shows the linear model, the gray area shows the confidence interval, N=205, p-value=0.462). (C) Cell counts according to lobar location (N=178, p-value=0.432). (D) Cortical cell counts according to hemisphere (N=164, p=0.745). (E) Cortical cell count according to sex (N=205, p-value=0.546). (F) Correlation of cortical cell counts with age (the blue line shows the linear model, the gray area shows the confidence interval, N=205, p-value=0.885)).*

***Supplementary Figure S4.*** *(A) Distribution of pTau isoforms among pTau only cases (N=39). (B) Propagation of Abeta deposits according to Thal phases. (C) Propagation of pTau deposits according to Braak & Braak stages. (D) Differences between males and females as a contingency table (N=205, p-value=0.262). (E) Effects of MGMT promoter methylation on ADNC as a contingency table (N=205, p-value=0.783). (F) Results of two independent neuropathological assessments for pTau and Abeta phenotypes (performed by SK and RR). pTau-related parameters in green, Abeta-related parameters in blue. (G) Logistic regression analysis for the impact on location and age on CERAD scores (N=106). (H) Logistic regression analysis for the impact on location and age on Braak & Braak stages (N=106). (I) Logistic regression analysis for the impact on location and age on Braak & Braak stages for patients younger than 60 years (N=80). (J) Frequencies of tumor locations in the whole cohort, displayed in a pie chart (N=205). (K) Frequencies of tumor locations for patients younger than 60 years, displayed in a pie chart (N=80). (L) Barplots depicting Abeta positive individuals in different studies of healthy individuals. (M) Barplots depicting pTau positive individuals in different studies of healthy individuals.*

***Supplementary Figure S5. Overall survival analysis.*** *(A) Kaplan Meier analysis for different age ranges (N=133, p-value=0.015). (B) Kaplan Meier analysis for ADNC for patients younger than 50 years (N=26, p-value=0.071). (C) Kaplan Meier analysis for ADNC for patients aged 50 to 75 years (N=70, p-value=0.58). (D) Kaplan Meier analysis for ADNC for patients older than 75 years (N=37, p-value=0.58). (E) Kaplan Meier analysis for CERAD scores for individuals aged 50-74 (N=70, p-value=0.30). (F) Kaplan Meier analysis for CERAD scores for individuals older than 75 (N=37, p-value=0.75). (G) Kaplan Meier analysis for Braak & Braak stages for individuals aged younger than 50 years (N=26, p-value=0.073). (H) Kaplan Meier analysis for Braak & Braak stages for individuals aged 50-74 years (N=70, p-value=0.30). (I) Kaplan Meier analysis for Braak & Braak stages for individuals aged older than 75 years (N=37, p-value=0.76). (J) Kaplan Meier analysis for ADNC for patients with methylated MGMT promoter state (N=72, p-value=0.034). (K) Kaplan Meier analysis for ADNC for patients with unmethylated MGMT promoter state (N=58, p-value=0.017). (L) Kaplan Meier analysis for Braak & Braak stages for patients with methylated MGMT promoter state (N=72, p-value=0.049). (M) Kaplan Meier analysis for Braak & Braak stages for patients with unmethylated MGMT promoter state (N=58, p-value=0.017). (N) Kaplan Meier analysis for CERAD scores for patients with methylated MGMT promoter state (N=72, p-value=0.61). (O) Kaplan Meier analysis for CERAD scores for patients with methylated MGMT promoter state (N=58, p-value=0.0097).*

***Supplementary Figure S6.*** *Microglia activation according to age and ADNC protein deposits. (A) Iba 1 counts according to age groups (N=205, p-value<0.0001). (B) Iba1 counts according to ADNC (N=205, p-value<0.0001).*

***Supplementary Figure S7.*** *Mapping of DIA (shown in red), particularly in and around necrosis (scale bar left = 1mm, scale bar right = 100µm).*

***Supplementary Table1.*** *Results of two different neuropathological assessments.*

| **Observation** | **Cohen´s Kappa** | **p-Value** |
| --- | --- | --- |
| Abeta | 0.701 | 2.28*10-7 |
| Core Plaques | 0.917 | 2.69*10-7 |
| Diffuse Plaques | 1 | 9.63*10-7 |
| CAA | 1 | 9.63*10-7 |
| pTau | 0.741 | 4.19*10-7 |
| NFTs | 0.673 | 4.82*10-4 |
| Pretangles | 0.814 | 4.94*10-5 |
| Threads | 1 | 9.63*10-7 |
| astrocytic | 1 | 9.63*10-7 |

***Supplementary Table2.*** *Treatment information for 8 patients with longitudinal samples.*

| **PatientID** | **Gender** | **Age at first surgery [years]** | **Time in between [months]** | **Surgery** | **Chemotherapy** | **Radiotherapy** | **ADNC-Status surgery 1** | **ADNC-Status surgery 2** |
| --- | --- | --- | --- | --- | --- | --- | --- | --- |
| 49 | m | 68 | 6.6 | subtotal resection | 1 | 1 | T | T |
| 51 | m | 79 | 0.2 | total resection | 1 | 1 | AT | T |
| 64 | f | 57 | 12.4 | total resection | 0 | 1 | none | T |
| 67 | m | 47 | 9.3 | total resection | 0 | 1 | none | none |
| 77 | m | 63 | 31.8 | subtotal resection | 0 | 0 | none | none |
| 86 | m | 67 | 10.5 | total resection | 1 | 1 | T | none |
| 88 | m | 52 | 13.7 | total resection | 1 | 1 | A | T |
| 118 | m | 74 | 10.4 | total resection | 1 | 1 | AT | AT |
