# Supplementary figures and images for: Frequent Alzheimer’s disease neuropathological change in patients with glioblastoma
Source: Neurooncol Adv. 2024 Jul 9;6(1):vdae118. doi: 10.1093/noajnl/vdae118 (PMC11362848; doi:10.1093/noajnl/vdae118)

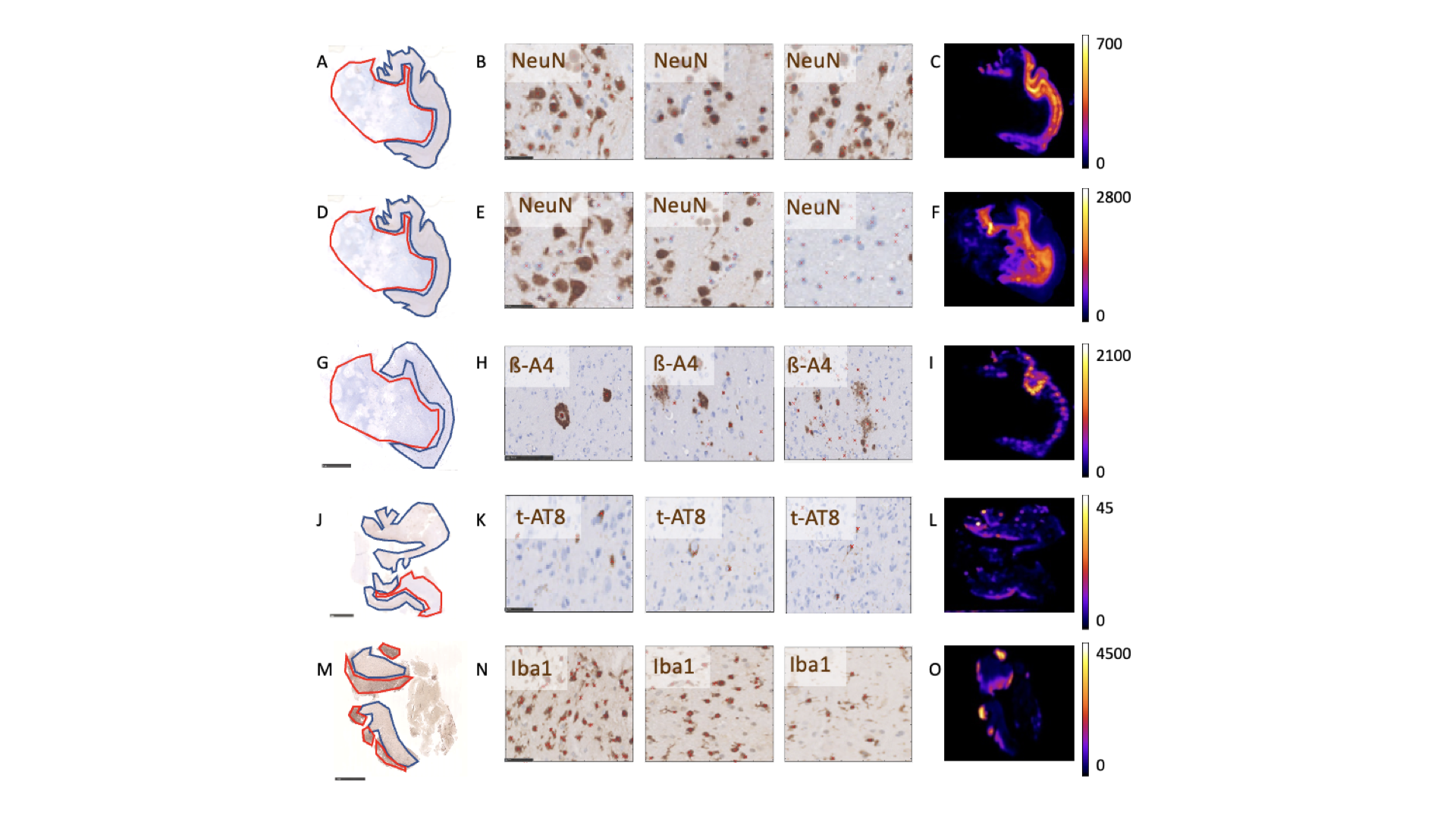

Supplement: vdae118_suppl_Supplementary_Data [file vdae118_suppl_Supplementary_Data.zip › renamed_289bb.tiff]

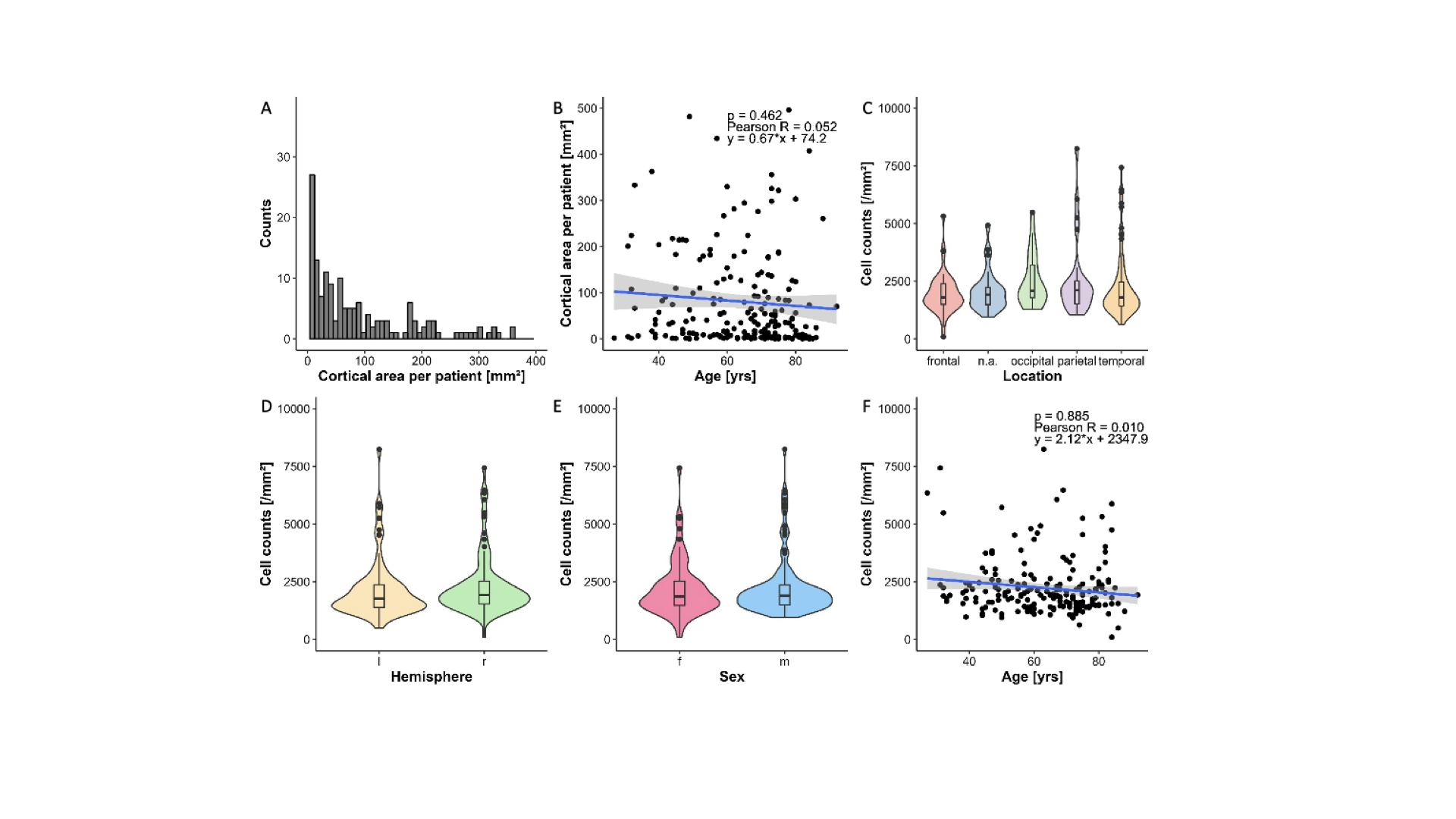

Supplement: vdae118_suppl_Supplementary_Data [file vdae118_suppl_Supplementary_Data.zip › renamed_56bfa.tiff]

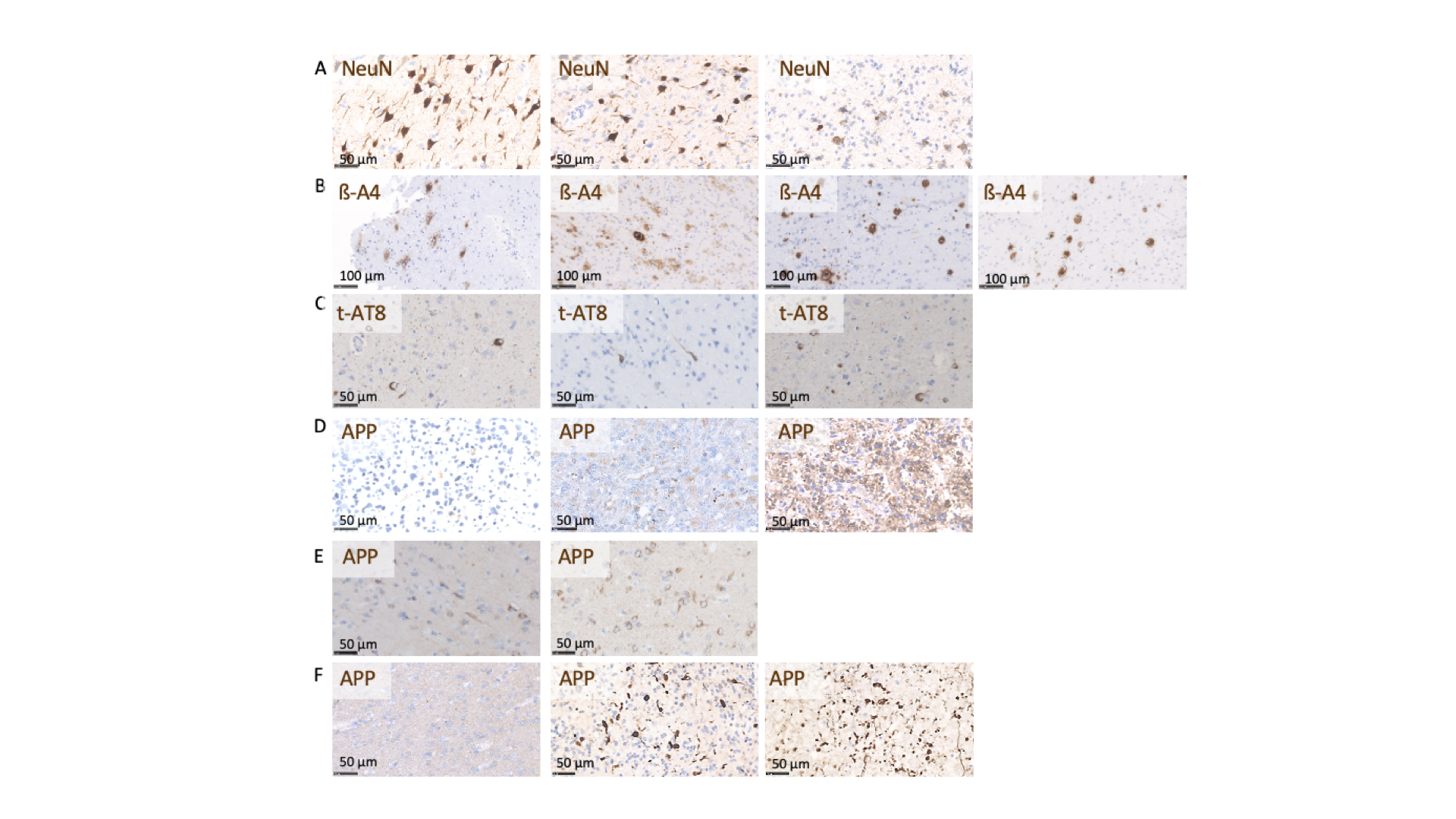

Supplement: vdae118_suppl_Supplementary_Data [file vdae118_suppl_Supplementary_Data.zip › renamed_6d4c0.tiff]

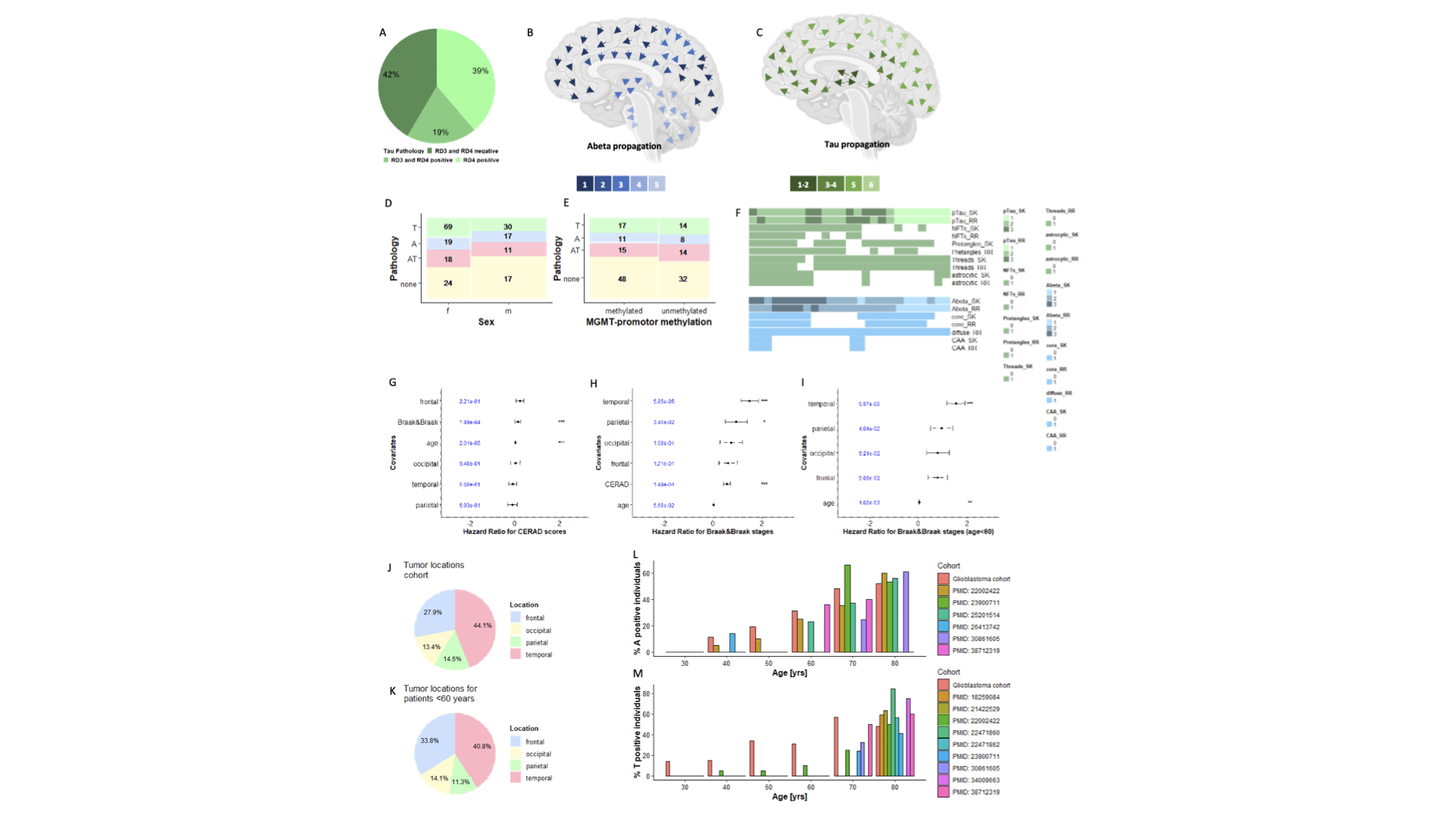

Supplement: vdae118_suppl_Supplementary_Data [file vdae118_suppl_Supplementary_Data.zip › renamed_910fd.tiff]

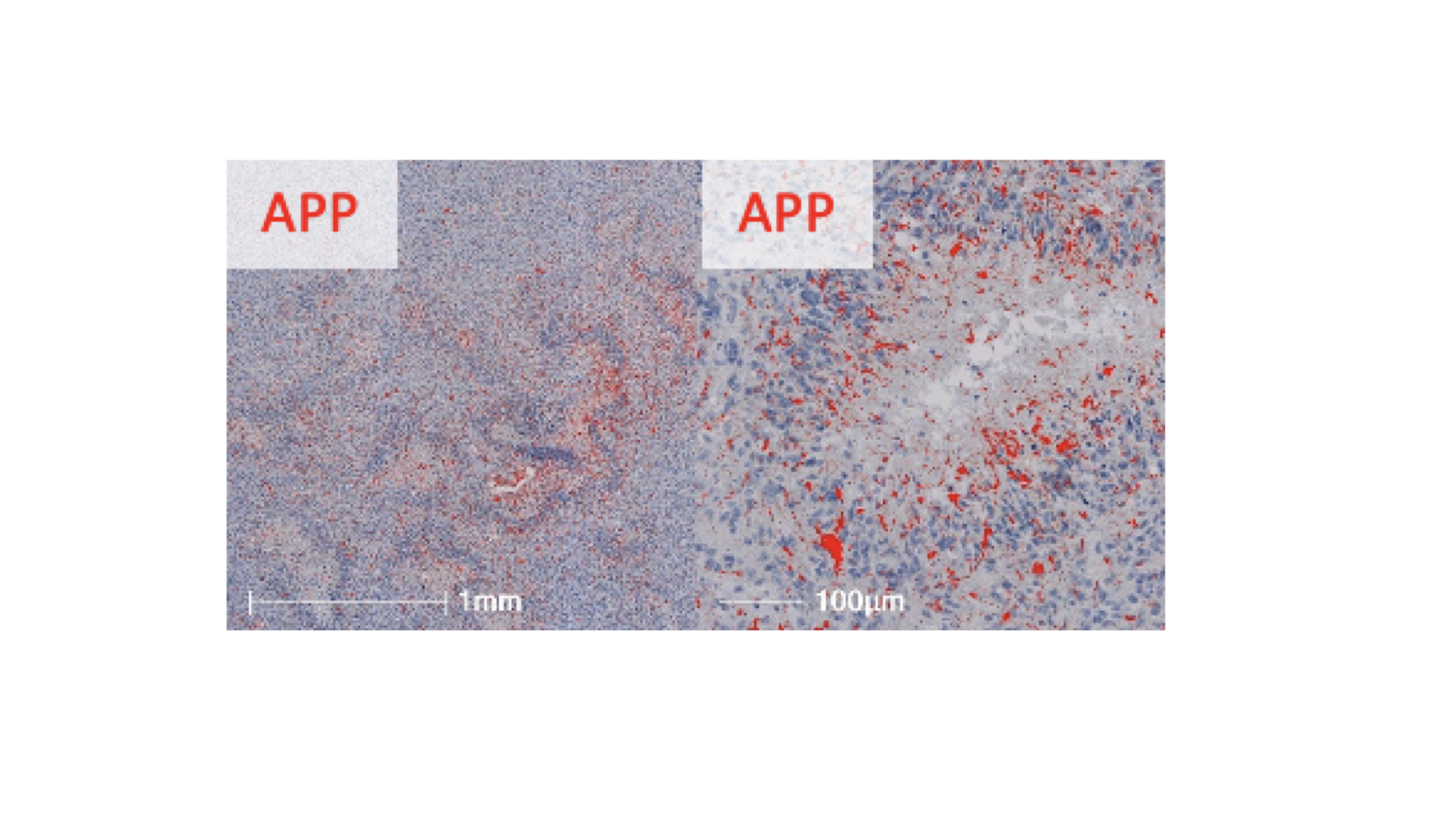

Supplement: vdae118_suppl_Supplementary_Data [file vdae118_suppl_Supplementary_Data.zip › renamed_ba7ce.tiff]

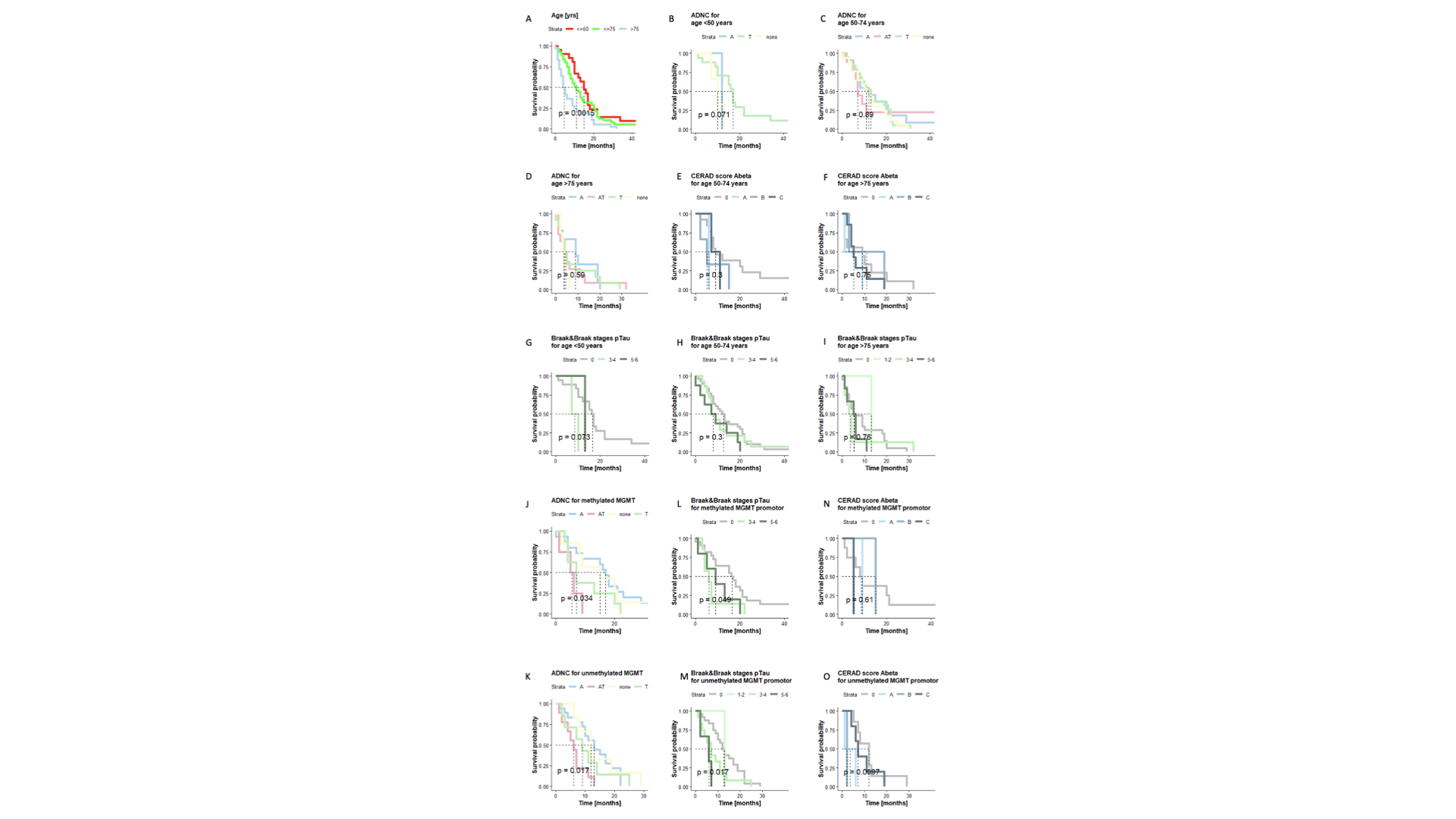

Supplement: vdae118_suppl_Supplementary_Data [file vdae118_suppl_Supplementary_Data.zip › renamed_c0282.tiff]

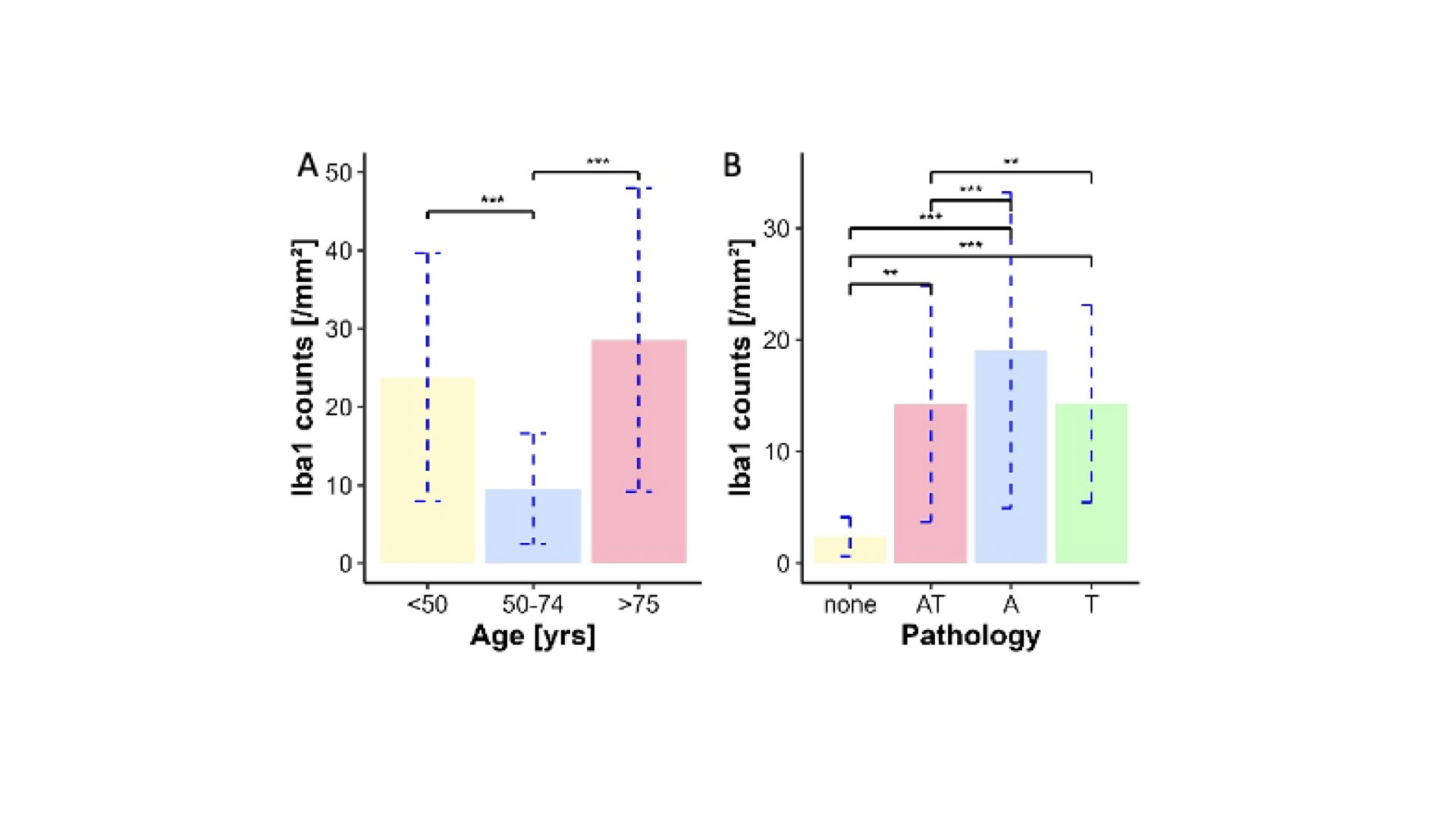

Supplement: vdae118_suppl_Supplementary_Data [file vdae118_suppl_Supplementary_Data.zip › renamed_f5464.tiff]
